# Rhino‐Orbital Mucormycosis in an Immunocompetent Adult: A Case Report Emphasizing Diagnostic and Therapeutic Challenges

**DOI:** 10.1002/ccr3.73683

**Published:** 2026-10-04

**Authors:** Seyed Mohammad Mousavi, Seyyed Amin Ayatollahi Mousavi, Omolbanin Faryabi, Iraj Sharifi, Hossein Kamyab, Zahra Shirazi

**Affiliations:** ^1^ Department of Medical Parasitology, Afzalipour School of Medicine, Research Center for Hydatid Disease in Iran, Institute of Infectious Diseases and Tropical Medicine Kerman University of Medical Sciences Kerman Iran; ^2^ Department of Medical Parasitology & Mycology, School of Medicine Kerman University of Medical Sciences Kerman Iran; ^3^ Leishmania Research Center Kerman University of Medical Sciences Kerman Iran; ^4^ Department of Neurosurgery, Prophet Azam Hospital Kerman University of Medical Sciences Kerman Iran; ^5^ Department of Infection Control, Prophet Azam Hospital Kerman University of Medical Sciences Kerman Iran

**Keywords:** amphotericin B, immunocompetent host, mucormycosis, occupational exposure, orbital exenteration, rhino‐orbital mucormycosis

## Abstract

Rhino‐orbital mucormycosis can present aggressively in immunocompetent adults, often with atypical initial symptoms that lead to misdiagnosis and dangerous delays. A high index of suspicion is paramount when facing a rapidly progressive orbital infection unresponsive to conventional therapy. Survival hinges on a prompt, aggressive multimodal strategy: immediate histopathological confirmation (identifying broad, aseptate hyphae), radical surgical debridement, and early initiation of high‐dose liposomal amphotericin B.

## 1 Introduction

Mucormycosis (formerly zygomycosis) is a rare, fatal opportunistic infection caused by environmental Mucorales molds [1, 2]. Infection occurs mainly via spore inhalation, and less commonly via trauma or ingestion [3, 4]. Rapid angioinvasion causes thrombosis and necrosis, driving fulminant progression with 40%–50% mortality despite treatment [2, 3]. Mucormycosis predominantly affects immunocompromised patients, notably those with diabetes, malignancies, or transplants [2, 3, 5]. Diabetes is the most common predisposing factor, with an overall mortality of approximately 46% [2]. However, cases in immunocompetent individuals are increasingly reported, often linked to agricultural occupational exposure [3]. Rhino‐orbital‐cerebral mucormycosis is the most common clinical form, followed by pulmonary and cutaneous types [2, 3]. ROCM spreads from the sinuses to the orbit and brain, causing proptosis, ophthalmoplegia, and vision loss [4, 6]. Hyperglycemia facilitates endothelial invasion, hyphal adhesion, and tissue damage [7, 8]. Novel virulence factors, such as mucoricin, contribute to vascular damage [9]. Diagnosis is challenging due to nonspecific symptoms and low culture sensitivity (30%–50%) [10]. Definitive diagnosis requires histopathological identification of broad, aseptate hyphae, confirmed by culture or molecular methods [10]. Although imaging (CT/MRI) findings are non‐specific, the presence of sinus opacification, orbital swelling, or fat stranding in a rapidly progressive setting should prompt early biopsy and histopathological evaluation [1]. Management involves surgical debridement, antifungal therapy, and correction of risk factors [1]. Liposomal amphotericin B is the first‐line antifungal agent [1]. Adjunctive therapies, such as posaconazole or isavuconazole for salvage, and hyperbaric oxygen or iron chelators in select cases, may enhance outcomes [1, 4]. Combined medical‐surgical approaches yield ~60% survival in ROCM [2, 6]. This case report describes an atypical presentation of rhino‐orbital mucormycosis (ROM) in an apparently immunocompetent greenhouse worker, highlighting occupational exposure as a risk factor, diagnostic pitfalls, and successful multimodal management.

## 2 Case History/Examination

A 30‐year‐old male greenhouse worker presented to his general practitioner with acute‐onset severe right‐sided epigastric pain and excessive epiphora (watering from the eye). He had no significant past medical, family, or psychiatric history and reported no history of diabetes mellitus, immunosuppressive therapy, or recurrent infections. His occupational history revealed prolonged daily exposure to humid, soil‐rich environments with decaying vegetation in a greenhouse setting.

Initial evaluation led to a provisional diagnosis of conjunctivitis, for which topical ketorolac eye drops were prescribed. Over the subsequent days, the patient's condition deteriorated, with persistent symptoms and the emergence of marked periorbital erythema progressing to ecchymosis around the affected eye. He was admitted to Prophet Azam Hospital, Kerman University of Medical Sciences, Kerman, Iran, for further evaluation and management.

On admission, the patient was alert and oriented, and afebrile. Ophthalmic examination revealed right periorbital erythema and ecchymosis, periorbital edema, and epiphora. Neurological examination showed no cranial nerve deficits and intact vision, and the remainder of the systemic examination was unremarkable. Given the rapidly progressive periorbital findings, the differential diagnosis at this stage included bacterial orbital cellulitis, cavernous sinus thrombosis, invasive fungal sinusitis (aspergillosis or mucormycosis), orbital pseudotumor, and malignancy (lymphoma or squamous cell carcinoma).

Non‐contrast computed tomography (CT) and magnetic resonance imaging (MRI) of the paranasal sinuses and orbits, performed on admission, revealed right‐sided proptosis with significant edema and infiltration of the intraconal and extraconal fat spaces. T2‐weighted MRI sequences demonstrated characteristic bright signal intensity within the affected orbital tissue, consistent with acute inflammatory changes (Figure 1a,b), with no definite evidence of intracranial extension or cavernous sinus thrombosis. Although these radiological findings are non‐specific and can also be seen in severe bacterial cellulitis or other inflammatory conditions, the combination of rapid clinical deterioration, extensive orbital involvement, and the patient's occupational history in a high‐risk environment raised suspicion for a fungal etiology. Imaging was instrumental in localizing disease extent, planning the surgical approach, and guiding the urgent biopsy that ultimately confirmed the diagnosis of mucormycosis.

**FIGURE 1:**
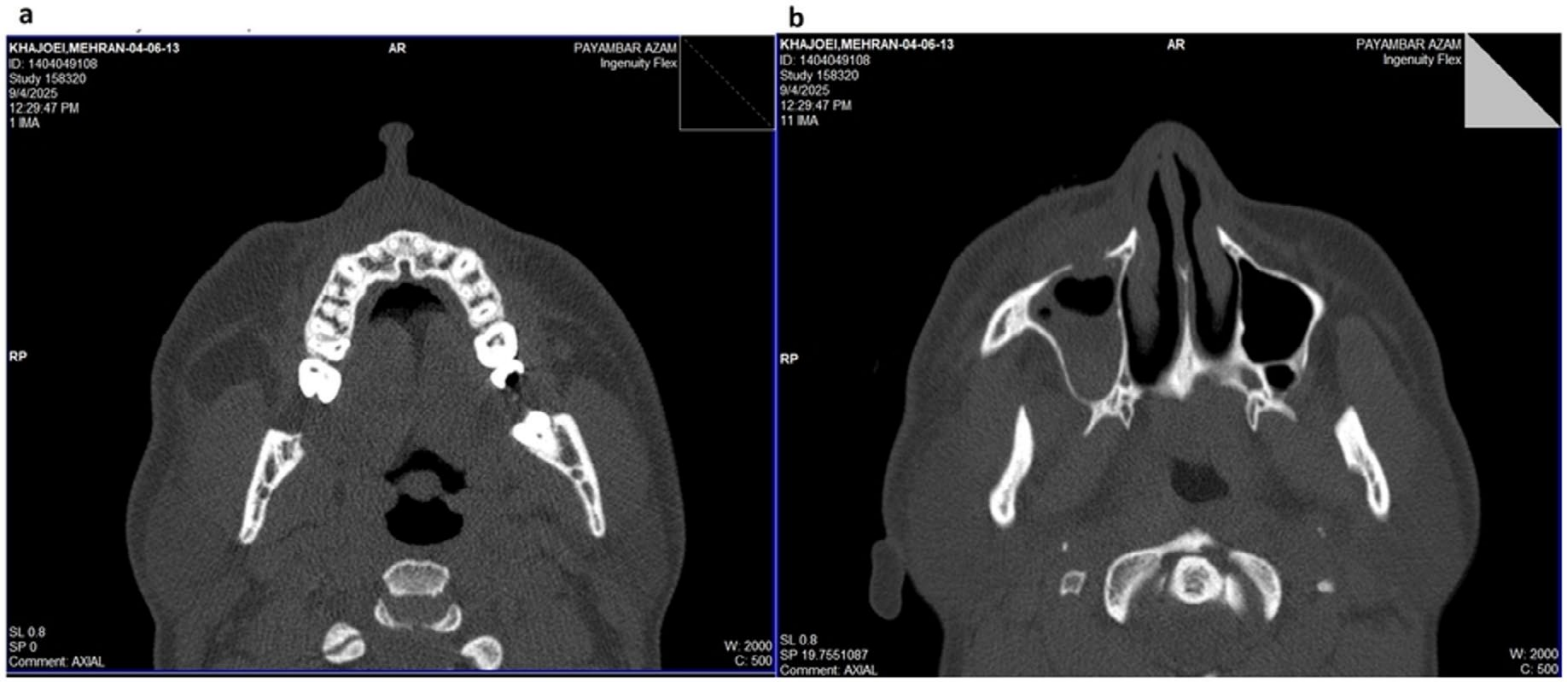
Orbital MRI findings at presentation. (a) T2‐weighted axial MRI demonstrating bright signal intensity within the right orbital extraconal fat, consistent with acute inflammatory changes and edema. (b) T2‐weighted coronal MRI showing significant infiltration of the intraconal and extraconal fat spaces with right‐sided proptosis. No definite evidence of intracranial extension or cavernous sinus thrombosis was observed.

Table 1 summarizes the key laboratory investigations performed during hospitalization, including a targeted immunological work‐up (HbA1c, HIV serology, and immunoglobulin levels) undertaken to evaluate for an occult immunocompromised state given the working classification of the patient as immunocompetent.

**TABLE 1:** Key laboratory findings.

Parameter	Value	Reference range
Hematology		
White blood cell count (WBC)	**12.6 × 10** ^**3**^ **/μL**	4.5–11.0 × 10^3^/μL
Neutrophils	**95%**	40%–75%
Lymphocytes	**5%**	20%–50%
C‐reactive protein (CRP)	**37 mg/L**	< 5 mg/L
Erythrocyte sedimentation rate (ESR)	**21 mm/h**	0–15 mm/h
Immunological work‐up		
HbA1c	5.3%	Prediabetes 5.7%–6.4%; Diabetes ≥ 6.5%
HIV serology (ELISA/Western blot)	Negative	Negative: < 1.0 S/CO; Positive: ≥ 1.0 S/CO
Total immunoglobulin G (IgG)	1200 mg/dL	Normal: 700–1600 mg/dL
Total immunoglobulin A (IgA)	340 mg/dL	Normal: 70–400 mg/dL
Total immunoglobulin M (IgM)	120 mg/dL	Normal: 40–230 mg/dL
Biochemistry		
Fasting blood glucose (FBS)	**110 mg/dL**	70–99 mg/dL
Alanine aminotransferase (ALT)	55 IU/L (initial); 47 IU/L (repeat)	7–56 IU/L
Alkaline phosphatase (ALP)	**224 U/L (initial); 220 U/L (repeat)**	44–147 U/L
Total bilirubin	**1.6 mg/dL**	0.1–1.2 mg/dL
Direct bilirubin	**0.7 mg/dL**	0–0.3 mg/dL
Renal function and electrolytes	Within normal limits	—

*Note:* Bold values indicate results outside the reference range. The immunological work‐up (HbA1c, HIV serology, immunoglobulins) was added to substantiate the classification of the patient as immunocompetent.

During drainage of eye discharge, necrotic tissue and purulent secretions were sampled and sent to the Department of Medical Parasitology & Mycology, Kerman University of Medical Sciences, for mycological analysis. Direct microscopic examination using potassium hydroxide (KOH) preparation, together with hematoxylin and eosin (H&E) and Giemsa staining, demonstrated broad, aseptate, ribbon‐like hyphae with wide‐angle branching consistent with Mucorales species (Figure 2a,b). Fungal culture on Sabouraud dextrose agar showed rapid, luxuriant growth after 7 days at 30°C, with fluffy, grayish‐white to brown colonies, confirming the diagnosis of mucormycosis (Figure 2c). The isolate was further identified to the species level as a *Rhizopus* species, based on macroscopic colony morphology and microscopic examination of a lactophenol cotton blue mount, which revealed broad, aseptate hyphae, sporangiophores with rhizoids at their base, and columella (Figure 2d). Repeat sampling from the skull base again yielded positive direct examination and culture results.

**FIGURE 2:**
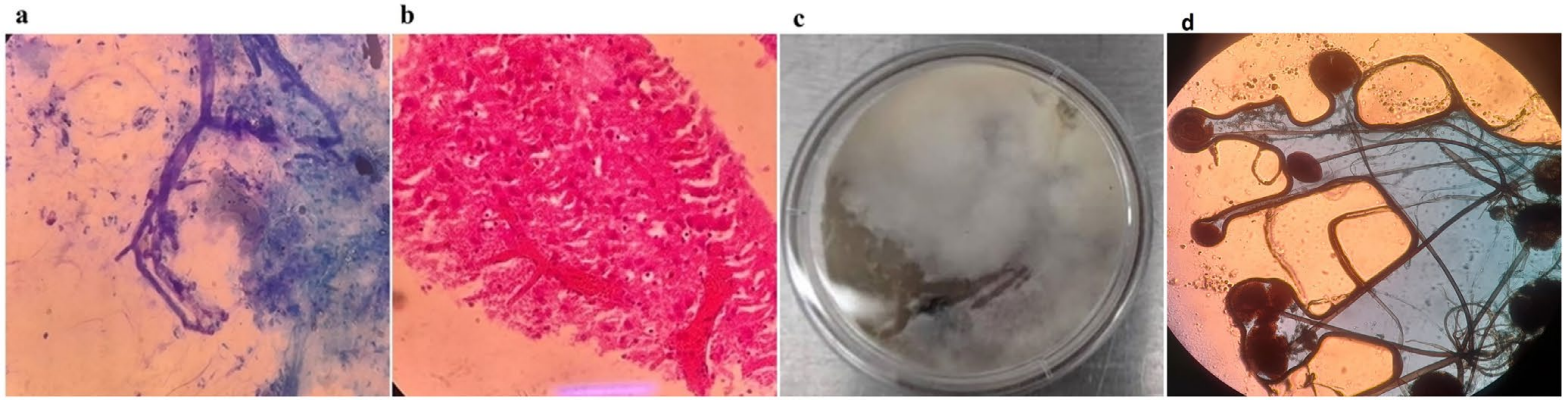
Mycological diagnosis of mucormycosis. (a) Direct microscopic examination of necrotic tissue using potassium hydroxide (KOH) preparation showing broad, aseptate, ribbon‐like hyphae with wide‐angle branching, characteristic of Mucorales species (original magnification ×400). (b) Hematoxylin and eosin (H&E) staining of tissue section demonstrating broad, aseptate hyphae with irregular branching invading necrotic tissue (original magnification ×400). (c) Fungal culture on Sabouraud dextrose agar after 7 days of incubation at 30°C, showing rapid, luxuriant growth with fluffy, grayish‐white to brown colonies. (d) Lactophenol cotton blue mount of the isolate revealing broad, aseptate hyphae, sporangiophores with rhizoids at their base, and columella, consistent with Rhizopus species (original magnification ×400).

Following histopathological confirmation of mucormycosis (Figure 3a), an aggressive multimodal therapeutic strategy was promptly initiated. The patient underwent urgent orbital exenteration to halt disease progression and prevent extension to the skull base; intraoperative findings revealed extensive necrotic tissue, underscoring the need for this radical debridement (Figure 3b,c). Concurrently, intravenous liposomal amphotericin B was administered at a dose of 5.0–10 mg/kg/day, continued for a total of 3 weeks.

**FIGURE 3:**
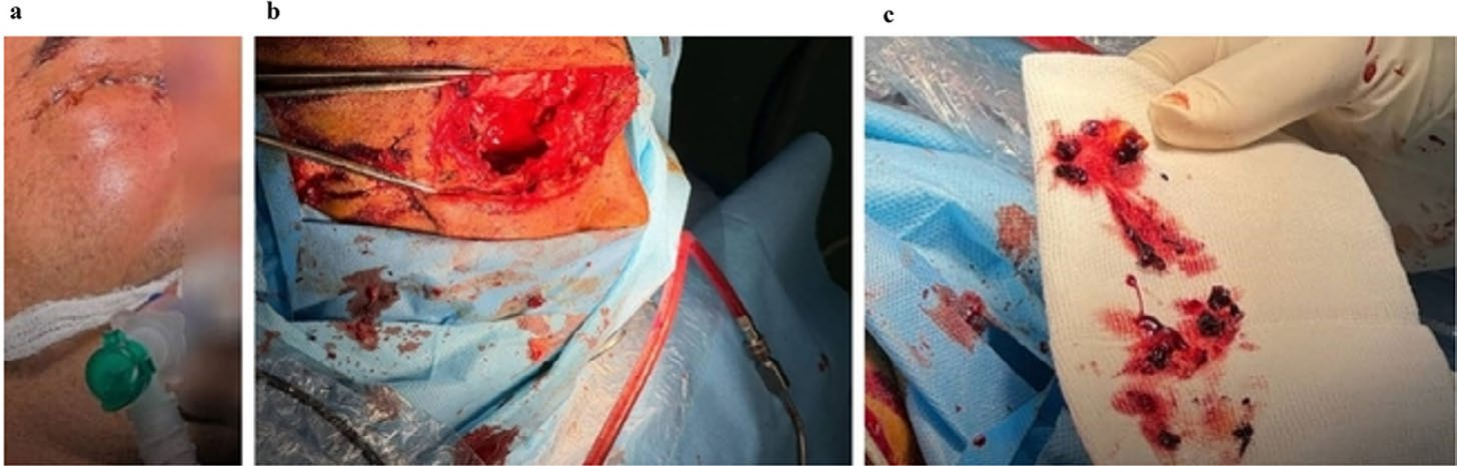
Intraoperative and clinical findings. (a) Patient's clinical presentation before surgery. (b) Intraoperative photograph showing extensive necrotic tissue involvement of the right orbit during orbital exenteration. (c) Surgical specimen following orbital exenteration, demonstrating extensive necrotic and devitalized tissue requiring radical debridement to prevent skull base extension.

This combined medical‐surgical approach was well tolerated, and the patient showed a favorable clinical response, culminating in discharge in stable condition upon completion of the antifungal course.

## 3 Conclusion and Results (Outcome and Follow‐Up)

The patient responded favorably to the combined medical and surgical approach. He was discharged in stable condition after completing 3 weeks of antifungal therapy. Final culture results corroborated the diagnosis 7 days post‐discharge.

At follow‐up assessment 3 months after discharge, the patient remained asymptomatic with no signs of recurrence or long‐term sequelae. He has been advised regarding occupational safety measures should he return to greenhouse work.

## 4 Discussion

This case exemplifies the diagnostic and therapeutic challenges of ROM in an apparently immunocompetent host, underscoring the role of occupational exposure as a predisposing factor. A central diagnostic challenge in this case was the atypical initial presentation: right‐sided epigastric pain and epiphora, without overt sinonasal or neurological signs, are highly unusual for ROM and can mislead even experienced clinicians, resulting in dangerous delays in diagnosis and treatment. Such atypical onset is not uncommon in ROM, where symptoms may mimic bacterial sinusitis or ocular infections, with progression to periorbital erythema, ecchymosis, and orbital involvement only later suggesting a fungal etiology [1, 4].

The clinical significance of the patient's initial epigastric pain warrants further consideration. While the primary route of infection in rhino‐orbital mucormycosis is spore inhalation, gastrointestinal acquisition through ingestion of contaminated material is also well documented [3, 4]. Given the patient's greenhouse occupation, with daily contact with soil and decaying vegetation, inadvertent ingestion of spores cannot be excluded. Gastrointestinal mucormycosis can itself present with abdominal pain, and early prodromal symptoms may occasionally precede overt sinonasal or orbital manifestations. Alternatively, the epigastric pain may have been an incidental, non‐specific finding that together with epiphora contributed to the initial misdiagnosis of conjunctivitis and the resulting delay in treatment. Regardless of its precise mechanism, this atypical presentation illustrates the diagnostic challenge posed by ROM in immunocompetent hosts, in whom vague or seemingly unrelated symptoms can obscure the underlying disease until orbital involvement becomes evident.

A key aspect of this case is the absence of traditional risk factors such as diabetes mellitus, hematological malignancy, or corticosteroid use, which are implicated in over 50% of mucormycosis cases in large‐scale reviews [2, 5]. This lack of overt immunosuppression likely contributed to the initially low index of suspicion for mucormycosis, underscoring that ROM can occur in healthy hosts, particularly those with environmental or occupational mold exposure. Instead, the patient's occupation as a greenhouse worker likely contributed to infection via inhalation of Mucorales spores, abundant in the humid, soil‐rich environments with decaying vegetation characteristic of greenhouse settings [3, 4].

Occupational exposures in agriculture have been linked to mucormycosis in several reports, although direct associations with greenhouse work specifically remain rare in the literature [11]. A cohort study of cucumber greenhouse workers documented substantial occupational exposure to airborne fungi, including Mucorales species, using next‐generation sequencing methods together with biomarkers of systemic inflammation supporting the biological plausibility of this exposure route, even though invasive disease was not observed in that cohort [11]. Comparable environmental risk has been described following natural disasters, such as a case of ROM in an immunocompetent adult after prolonged mold exposure during post‐hurricane cleanup [12], and rural living or contact with organic matter has similarly been implicated in pediatric orbital mucormycosis without underlying immunodeficiency [13]. To our knowledge, the present case is among the few reports to link ROM directly to greenhouse‐based occupational exposure, reinforcing that high‐inoculum environmental exposure rather than host immunosuppression alone can be sufficient to precipitate invasive disease. Such exposure may overwhelm innate defenses, particularly phagocytosis by neutrophils and macrophages, which are typically effective against Mucorales [8].

The patient's impaired fasting glucose (110 mg/dL) represents a subtle metabolic derangement that may have facilitated pathogenesis. Even mild hyperglycemia upregulates endothelial receptors such as GRP78, enhancing fungal adhesion and angioinvasion [7, 8], consistent with emerging evidence that transient or subclinical dysglycemia can predispose otherwise healthy individuals to opportunistic mycoses [4]. Laboratory findings, including leukocytosis with neutrophilia and elevated inflammatory markers (CRP 37 mg/L, ESR 21 mm/h), reflected a robust systemic immune response that was nonetheless insufficient to contain the infection without aggressive intervention. Formal immunological work‐up HbA1c, HIV serology, and immunoglobulin levels (Table 1) did not identify an underlying immunocompromised state, supporting the classification of this patient as immunocompetent and reinforcing that high‐inoculum environmental exposure alone was sufficient to precipitate invasive disease.

Diagnosis relied on histopathological demonstration of broad, aseptate hyphae with right angle (90°) branching, a pathognomonic feature confirmed by KOH preparation and staining [1]. Although culture on Sabouraud dextrose agar subsequently yielded rapid growth in this case, treatment was initiated on the basis of microscopy alone, a critical approach given the generally low sensitivity (30%–50%) and slow turnaround of fungal culture [10]. Species‐level identification of the isolate as a *Rhizopus* species, achieved through colony morphology and lactophenol cotton blue microscopy, further supported targeted management; however, such identification is time‐consuming and should never delay empirical antifungal therapy. Direct microscopy and histopathological examination of biopsy specimens therefore remain the cornerstones of rapid diagnosis in the clinical setting, reinforcing that therapeutic decisions should be made on histopathological grounds alone [7].

Management followed established guidelines, emphasizing surgical debridement and antifungal therapy [1]. Orbital exenteration, though radical, prevented cerebral extension, which carries > 80% mortality [14]. Liposomal amphotericin B (5.0–10 mg/kg/day for three weeks) was effective, leveraging its superior tolerability and activity against Mucorales [1]. Outcomes in ROM vary, with combined medical‐surgical approaches yielding ~60% survival, compared with > 90% mortality in disseminated disease [2, 14]. In immunocompetent hosts, prognosis appears more favorable, as systematic reviews show lower mortality when underlying predisposing conditions are absent [3].

To contextualize, a literature search across PubMed, Scopus, and Web of Science (January 2000–October 2025) using terms such as “rhino‐orbital mucormycosis immunocompetent” and “mucormycosis occupational exposure” identified relevant case reports; key similar cases are summarized in Table 2.

**TABLE 2:** Summary of selected case reports on rhino‐orbital mucormycosis with an occupational exposure.

References	Patient Age/Sex	Risk Factors/Exposure	Presentation	Diagnosis	Treatment	Outcome
Ayoade et al. (2019) [12]	67/F	Post‐hurricane mold cleanup	Headache, diplopia, vision loss	Histopathology (pauci‐septate hyphae)	Multiple debridements, amphotericin B	Full recovery, no recurrence at 6 months
Razem et al. (2021) [15]	59/F	None identified	Periorbital edema, vision loss, nasal obstruction	Post‐mortem histopathology	Extensive surgical resection	Death due to multiple organ failure
Taha et al. (2018) [14]	32/M	None identified	Headache, fever, weakness, seizure	Biopsy (aseptate hyphae)	Amphotericin B + voriconazole	Death due to non‐compliance
Prado‐Calleros et al. (2021) [16]	12/F	Foreign body in nose	Rhinosinusitis, orbital abscess, diplopia	KOH, PCR (Rhizopus oryzae)	Surgery, amphotericin B	Survival, asymptomatic at 6 months
Dhiyantari et al. (2024) [17]	22/M	Minor skin lesion	Orbital cellulitis, abscesses, proptosis	CT, PAS staining	Exenteration, amphotericin B + itraconazole	No recurrence at 6 months
Amanati et al. (2020) [13]	Mean 16 months (F/M) (5 cases)	Rural living, hay contact, trauma	Periorbital swelling, proptosis	Histopathology, culture, PCR	Amphotericin B, debridement	All cured, no mortality

Across these reports, diagnostic delay emerges as a recurring theme in immunocompetent patients, often driven by the absence of classic risk factors and the non‐specific nature of early symptoms underscoring the need for greater awareness among primary care physicians and emergency clinicians [7, 9]. Unlike diabetic hosts, angioinvasion in immunocompetent patients may follow a somewhat less fulminant course, allowing a window for recovery [7, 9]. However, as illustrated by a report in which death resulted from treatment noncompliance, favorable outcomes still depend critically on adherence to therapy [14].

In conclusion, this case reinforces that ROM should be considered in progressive orbital infections, particularly with occupational exposures, even in the absence of overt immunodeficiency. High clinical suspicion, prompt histopathological diagnosis, and aggressive multimodal therapy are pivotal for survival.

## Author Contributions

**Seyed Mohammad Mousavi:** conceptualization, formal analysis, visualization, writing – original draft, writing – review and editing. **Omolbanin Faryabi:** conceptualization, validation, writing – original draft, writing – review and editing. **Seyyed Amin Ayatollahi Mousavi:** writing – original draft, writing – review and editing. **Iraj Sharifi:** supervision, visualization, writing – original draft, writing – review and editing. **Hossein Kamyab:** data curation, project administration. **Zahra Shirazi:** conceptualization, supervision, visualization, writing – original draft, writing – review and editing.

## Funding

The authors have nothing to report.

## Consent

Written informed consent for publication of this case report and accompanying images was not obtained from the patient as he has been lost to follow‐up and cannot be contacted despite reasonable efforts. All identifying information has been removed to ensure patient anonymity.

## Conflicts of Interest

The authors declare no conflicts of interest.

## Data Availability

All relevant data are included within the article and its tables and figures.
